# Food from the Depths of the Mediterranean: The Role of Habitats, Changes in the Sea-Bottom Temperature and Fishing Pressure

**DOI:** 10.3390/foods11101420

**Published:** 2022-05-13

**Authors:** Porzia Maiorano, Francesca Capezzuto, Angela Carluccio, Crescenza Calculli, Giulia Cipriano, Roberto Carlucci, Pasquale Ricci, Letizia Sion, Angelo Tursi, Gianfranco D’Onghia

**Affiliations:** 1 Department of Biology, University of Bari Aldo Moro, Via E. Orabona 4, 70125 Bari, Italy; porzia.maiorano@uniba.it (P.M.); angela.carluccio@uniba.it (A.C.); giulia.cipriano@uniba.it (G.C.); roberto.carlucci@uniba.it (R.C.); pasquale.ricci@uniba.it (P.R.); letizia.sion@uniba.it (L.S.); angelo.tursi@uniba.it (A.T.); gianfranco.donghia@uniba.it (G.D.); 2 Consorzio Nazionale Interuniversitario per le Scienze del Mare (CoNISMa), Piazzale Flaminio 9, 00196 Roma, Italy; 3 Department of Economy and Finance, University of Bari Aldo Moro, Largo Abbazia S. Scolastica, 70124 Bari, Italy; crescenza.calculli@uniba.it

**Keywords:** seafood, fisheries resources, vulnerable marine ecosystem, environmental change, conservation, Mediterranean

## Abstract

As part of the “*Innovations in the Food System: Exploring the Future of Food*” Special Issue, this paper briefly reviews studies that highlight a link between deep-sea fishery resources (deep-sea food resources) and vulnerable marine ecosystems (VME), species, and habitats in the Mediterranean Sea, providing new insights into changes in commercial and experimental catches of the deep-sea fishery resources in the central Mediterranean over the last 30 years. About 40% of the total landing of Mediterranean deep-water species is caught in the central basin. Significant changes in the abundance of some of these resources with time, sea-bottom temperature (SBT), and fishing effort (FE) have been detected, as well as an effect of the Santa Maria di Leuca cold-water coral province on the abundance of the deep-sea commercial crustaceans and fishes. The implications of these findings and the presence of several geomorphological features, sensitive habitats, and VMEs in the central Mediterranean are discussed with respect to the objectives of biodiversity conservation combined with those of management of fishery resources.

## 1. Introduction

Seafood is a fundamental source of proteins and nutrients for human nutrition. Its global consumption has increased since 1960 at rates per year higher than that of all land animals, combined and individually (i.e., bovine, ovine, porcine, etc.), except for poultry [1]. However, with the increasing demand for sea products, most of the fishery resources of the world’s oceans have been overfished, and many are in a condition of variable levels according to the vulnerability of the different species. In addition, the overfishing condition of continental shelf fish resources has pushed the fishing activities to move towards the exploitable living resources of the deep sea [2,3,4]. In this regard, deep waters (i.e., beyond the continental shelf, and deeper than approximately 200 m) have acted as a refuge for several stocks with an extensive vertical distribution, where no fishing was occurring until the first decades of the last century [5]. With the expansion of fishing to deeper waters, favoured by the development of new technologies, muddy deep bottoms and other deep-water refuges—such as soft-bottom coral gardens (CGs), cold-water coral (CWC) reefs, submarine canyons (SCs), and seamounts (SEs)—have been affected by this activity, and may no longer play their role of providing structure, food, and shelter for fishery resources. All of these deep-water ecosystems have been identified as hotspots of biodiversity (e.g., [6,7,8]), and since they can be valuable fishing sites, due to the occurrence of large sizes and high abundances of commercial species, they are often impacted by commercial fishing [9,10,11,12,13,14,15,16,17,18,19,20]. Fisheries for some deep-sea fish stocks have also collapsed due to heavy pressure on species whose life history traits do not make them well-suited to an intensive harvest [3,4,21]. In fact, deep-sea fish species have a longer lifespan, slow growth, and later sexual maturity, and are consequently more vulnerable and less resilient to overfishing [22,23,24,25,26,27].

The EU began to deal with deep-sea fisheries in 1992, as a result of assessments carried out by the International Council for the Exploration of the Sea (ICES), who stated that most of the deep-water species of commercial interest were overfished [28]. The current scientific evidence suggests that many deep-sea fish stocks are being exploited beyond sustainable levels [23,29,30,31,32], thus emphasising the need to improve the management of these species [3,32,33,34,35].

It is well-known that fishing can impact harvested populations directly by excessive removal of individuals, and indirectly by reducing habitat complex structures that guarantee their bioecological activities and provide protection from predators, as well as from adverse physical factors (e.g., [36,37,38,39,40]). Globally, around 40% of trawling fishing grounds are on waters deeper than the continental shelf [41], and several deep-sea habitats and ecosystems have been impacted by fishery activities, with consequent depletion of or reduction in economically important species (e.g., [3,9,21,42,43,44,45]).

According to the FAO [46], vulnerable marine ecosystems (VMEs) are groups of species, communities, or habitats that may be vulnerable to impacts from fishing activities. Corals, together with sponges, echinoderms, molluscs, and other epibenthic species, play a significant role in the formation of VMEs. Furthermore, most coral taxa are included in relevant lists of protected species, such as the Red List drawn up by the International Union for the Conservation of Nature (IUCN) [47,48,49,50]. Their vulnerability is linked to their likelihood of experiencing substantial alterations from short-term or chronic disturbances, as well as to their difficulty in recovering (for example, slow growth rate, late age of maturity, low or unpredictable recruitment, and long life expectancy) [51,52,53,54,55]. In addition, there is sufficient evidence that VMEs act as essential fish habitats (EFHs), defined as “those waters and substrates necessary to fish for spawning, breeding, feeding, or growth to maturity” [56,57,58]. Therefore, there is an international consensus in favour of the protection of VMEs in order to combine conservation and fisheries-management objectives according to the ecosystem approach to fisheries (EAF) [46,59,60,61,62,63,64].

Fish consumption has always been an important part of people’s diets around the Mediterranean Sea. The annual production of about 788,000 tonnes, for a total revenue of USD 3.4 billion, offers employment opportunities to several hundred thousand people, supplies seafood products for human consumption to local and regional markets, and creates many other indirect benefits, although fisheries and related activities also produce a large amount of marine litter (including plastics) as a global increasing threat [1]. In the Mediterranean Sea the exploitation of deep-water resources only started in the first decades of the last century. In particular, the deep-water red shrimps *Aristaeomorpha foliacea* (giant red shrimp) and *Aristeus antennatus* (blue and red shrimp) were the target species for deep-water bottom trawling in the 1930s in the Ligurian Sea, where catches of these two shrimps were between 100 and 200 kg/day and, after the Second World War, they could be up to 1000 kg/day per boat [65,66]. In the 1940s, these resources began to be exploited in the Catalan and Balearic seas, and subsequently in other Mediterranean areas [67]. In the Mediterranean Basin, the development of deep-water commercial fisheries was due to the narrowness of the shelf, crossed by several submarine canyons, as well as the growth of the human population along the basin and high demand for a much-appreciated food. However, considering the multispecies nature of the Mediterranean fisheries, the deep-water red shrimps are mostly caught in bathyal muddy bottoms, between 400 and 800 m, together with many other valuable demersal species, such as the European hake (*Merluccius merluccius*), the deep-water rose shrimp (*Parapenaeus longirostris*), the Norway lobster (*Nephrops norvegicus*), the blackspot seabream (*Pagellus bogaraveo*), the greater forkbeard (*Phycis blennoides*), and the wreckfish (*Polyprion americanus*). Other deep-water species of commercial interest, such as the blue whiting (*Micromesistius poutassou*), European conger (*Conger conger*), blackbelly rosefish (*Helicolenus dactylopterus*), angler (*Lophius piscatorius*), blackbellied angler (*Lophius budegassa*), blackmouth catshark (*Galeus melastomus*), bluntnose sixgill shark (*Hexanchus griseus*), golden shrimp (*Plesionika martia*), southern shortfin squid (*Illex coindetii*), European flying squid (*Todarodes sagittatus*), and lesser flying squid (*Todaropsis eblanae*), represent additional components of deep catches. Most of the fish species are commonly caught with trawl nets, longlines, and gillnets, while crustaceans and cephalopods are fished mainly with trawl nets, and sometime with pots.

Most of the abovementioned species are distributed across a wide depth range, between shelf and slope; some of them are typically distributed in deep waters. Moreover, past and recent investigations in deep-sea habitats with a complex and heterogeneous structure—such as coral ecosystems, submarine canyons, and seamounts (now considered VMEs)—have proven that most of the abovementioned species use these types of habitats for shelter, feeding, spawning, and nursery (e.g., [20,58,68,69,70,71]). Indeed, habitat features play an important role in determining the structure of species assemblages, and habitat loss may impact—albeit in different ways—on all life stages and critical phases of the different species [38,72]. While adult individuals may not be strictly affected by habitat as juveniles, the detrimental effects of habitat loss on juvenile survival may have longer-term impacts on adult populations [73].

The innovation and sustainability of the food (in the present case, the fishery resources represented by deep-sea fish, crustaceans, and molluscs) used by humanity are mainly based on the systems where it is produced (i.e., the deep-sea sensitive habitats and VMEs), and on the systems that allow its harvest (i.e., the fishing techniques). As part of the “*Innovations in the Food System: Exploring the Future of Food*” Special Issue, this paper aims (1) to briefly review studies that highlight a link between deep-sea fishery resources and VMEs in the Mediterranean Sea; (2) to provide new insights into commercial and experimental catches of the deep-sea fishery resources in the central Mediterranean for the past 30 years; (3) to evaluate changes in the abundance of these resources with time, sea-bottom temperature (SBT), fishing effort (FE), and depth; and (4) to reveal an effect of the Santa Maria di Leuca cold-water coral province on the abundance of the deep-sea fishery resources. The implications of these findings and the presence of several geomorphological features, sensitive habitats, and VMEs in the central Mediterranean are discussed in terms of conservation of biodiversity, combined with the sustainable management of the fishery resources.

## 2. Materials and Methods

Data on food resources from deep-sea sensitive habitats and VMEs in the whole Mediterranean basin refer to international volumes and publications, and references therein (e.g., [11,55,67,68,74,75,76,77]).

**Figure 1:**
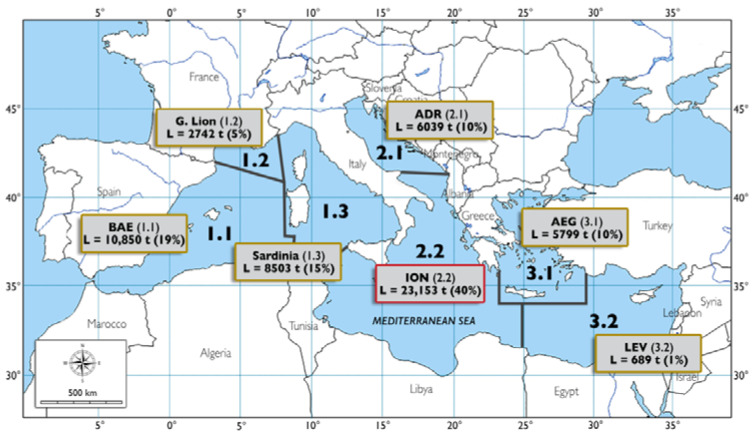
Average total landing (L) of deep-sea commercial species for each FAO Mediterranean subdivision, expressed in tonnes (t) and percentages (%), calculated for the period 1994–2019. FAO subdivisions are coded as Balearic (BAE, 1.1), Gulf of Lion (G. Lion, 1.2), Sardinia (SAR, 1.3), Adriatic (ADR, 2.1), Ionian (ION, 2.2), Aegean (AEG; 3.1), and Levant (LEV, 3.2).

In order to define the contribution of the most important commercial deep-sea species as food resources from the depths of the Mediterranean Sea, landing data from official FAO statistics were explored (https://www.fao.org/fishery/statistics-query/en/gfcm_capture/gfcm_capture_quantity, accessed on 12 January 2022), focusing on the central part of the basin (Figure 1).

The temporal trends over a period of 25 years (1994–2019) were analysed by means of Spearman’s non-parametric correlation.

New observations from the central Mediterranean, the southwestern Adriatic, and the northwestern Ionian, as well as on muddy bottoms of the northwestern Ionian Sea, were derived from data collected as part of national and international study projects carried out in the last two decades by the ecology team from the Department of Biology at the University of Bari Aldo Moro. In particular, data from deep-sea sensitive habitats and VMEs were taken using different low-impact fishing techniques [12,71,78,79,80,81,82,83,84,85], while data from muddy bottoms were collected during the Mediterranean Trawl Surveys (MEDITS) programme, included in the EU Data Collection Framework to date [86]. The MEDITS surveys are carried out in the Mediterranean from late spring to summer every year, according to a standardised protocol that includes gear characteristics, haul duration, and sampling procedures, following a depth-stratified random design, from 10 to 800 m in depth [86,87,88,89].

**Figure 2:**
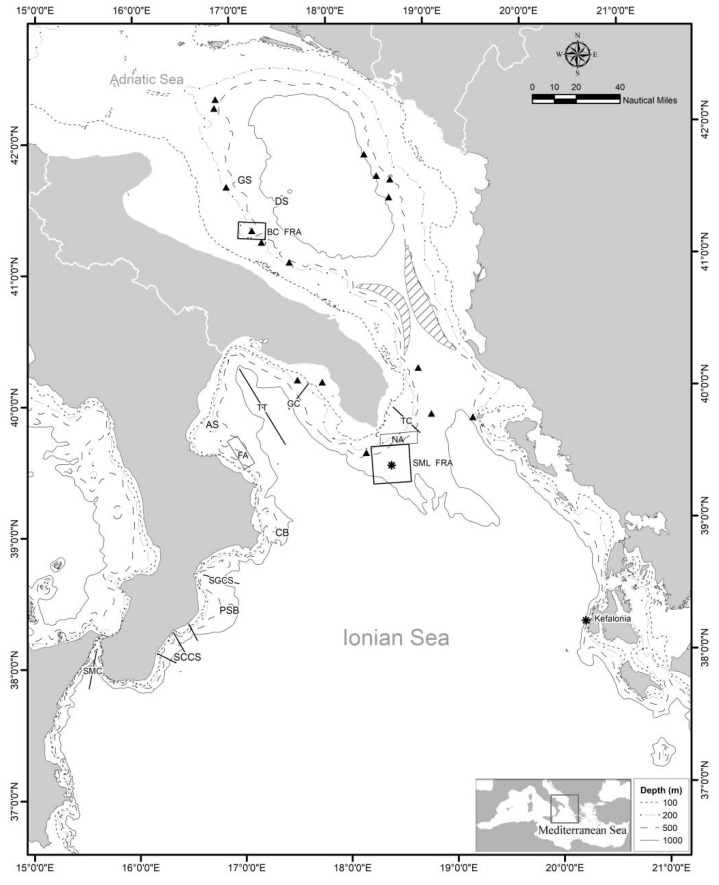
Topographic features, highs, banks, canyons, sensitive habitats, and VME species and habitats along the southwestern Adriatic Sea and northwestern Ionian Sea (central Mediterranean). GS = Gondola Slide; BC FRA = Bari Canyon Fisheries Restricted Area; SML FRA = Santa Maria di Leuca Fisheries Restricted Area; TC = Tricase Canyon; GC = Gallipoli Canyon; TT = Taranto Trench; AS = Amendolara Shoal; CB = Crotone Bank; SGCS = Squillace Gulf Canyon System; PSB = Punta Stilo Bank; SCCS = South Calabria Canyon System; SMC = Strait of Messina Canyon; triangle = hard bottom corals; asterisk = hard- and soft-bottomed corals [58,81,90,91,92,93,94,95,96,97]; lined areas *= Isidella elongata* facies [98]; NA = sampling area near the SML FRA; FA = sampling area far from the SML FRA.

Using MEDITS data, the abundances in weight (expressed as biomass index kg/km^2^) and numbers (expressed as density index N/km^2^) of the deep-sea species distributed on deep muddy bottoms (200–800 m) of the northwestern Ionian Sea (Figure 2) were evaluated for the period 1994–2020, and their changes over time were tested using Spearman’s non-parametric correlation. Data on the sea-bottom temperature (SBT) were recorded using a probe at the start and the end of different MEDITS hauls carried out from 1998 to 2020, and an average value of SBT was computed per year. The fishing pressure of bottom trawling fleets on the resources of the northwestern Ionian Sea was analysed using fishing effort (FE) data, in terms of number of vessels and gross tonnage (GT). Data were obtained from the European Fishing Fleet Register (http://ec.europa.eu/fisheries/fleet/index.cfm, accessed on 12 January 2022) for the period 1994–2020. The relationship between abundance of the deep-sea species and SBT was evaluated using linear regression analysis. Spearman’s non-parametric correlation was also applied to the abundance data in weight (kg/km^2^) of the deep-sea species that showed a significant trend over time versus the FE—expressed as the total annual number of vessels—throughout the study period.

Furthermore, for the shrimps *A. foliacea* and *P. longirostris*, due to correlation between environmental covariates (*ρ* = −0.73, *p* < 0.001), and in order to avoid multicollinearity problems, linear regression models were estimated to investigate the dependence between log-transformed abundances and environmental drivers (i.e., SBT and FE). Transformed responses ensure, in these cases, that the model assumptions are met. For both species, ***s*** = {*A. foliacea*, *P. longirostris*}, and for each ***y_z_*** with ***z*** = {abundance in weight, abundance in number}, the linear model is specified as follows:

(1) $$log(y_{z}^{\left(s\right)})=\beta_{0}^{\left(s\right)}+\beta_{1}^{\left(s\right)}x_{i}^{\left(s\right)}+\epsilon^{\left(s\right)}$$

where ***x_i_*** represents the independent covariate, with ***i*** = {FE, SBT}.

Using linear regression analysis, the relationship between length and depth was evaluated for *M. merluccius*, *P. bogaraveo*, *P. blennoides*, *H. dactylopterus*, and *Galeus melastomus*, collected both on muddy bottoms with the MEDITS trawl net and in VMEs with an experimental longline (e.g., [81]), and the boxplots of the length were represented for all of these species in both habitats.

In order to detect an effect of the presence of a VME on fishery resources, MEDITS abundance data on the weight and number of the species *A. foliacea*, *P. martia*, *M. merluccius*, *P. bogaraveo*, *P. blennoides*, and *H. dactylopterus*, for an area near the Santa Maria di Leuca (SML) cold-water coral (CWC) province (NA), were compared with those of other species far from this coral province (FA) (Figure 2). Data from 54 trawl hauls, carried out between 200 and 800 m, were used for each area. Relative boxplots of the abundances in weight and number were produced, and the differences in the abundances between the two areas were tested using the Kruskal–Wallis non-parametric test. The pressure of fishing activity in these two areas was assessed in order to exclude effects due to this activity on the results of the comparison between the two areas. Specifically, FE was calculated for NA and FA by aggregating the fleets operating close to the two areas. In particular, the fleets of Gallipoli, Leuca, and Otranto were considered for NA, while those of Corigliano Calabro and Cirò Marina for FA. The differences in the species abundances between the two areas were tested using the Kruskal–Wallis non-parametric test.

## 3. Review of the Mediterranean Studies on the Link between Deep-Sea VMEs and Fishery Resources

In the Mediterranean Sea, the deep-water resources are mainly exploited by trawl fishing on the soft bottoms of the bathyal grounds. However, there are areas characterised by the occurrence of VME species on soft and hard bottoms, canyons, and seamounts, where the fishing is carried out using different types of gears.

### 3.1. Open Slope, Soft Bottoms

Between the shelf break and descent to bottoms deeper than 1000 m, soft corals can be found that can form dense aggregations on soft bottoms—called sea pen fields, sea fan corals, and arborescent corals—which build up coral gardens or coral forests. Coral gardens can develop on soft or hard substrata, depending on the habitat-forming species. Both sea pen fields and coral gardens and/or coral forests contribute to making more heterogeneous and complex habitats, attracting mobile and swimming fauna [55].

The sea pen fields built up by the octocoral *Funiculina quadrangularis* are mostly distributed on the upper slope, generally at less than 400 m in depth, on soft muddy habitats characterised by noticeable bottom currents. These habitats are commonly inhabited by commercially valuable crustaceans, such as the deep-water rose shrimp (*P. longirostris*) and the Norway lobster (*N. norvegicus*). Soft-bottomed coral gardens, structured by the gorgonian *Isidella elongata* (bamboo coral), can be found from the shelf break down to 1600 m. The valuable deep-water red shrimps (*A. antennatus*, *A. foliacea*) and the golden shrimp (*P. martia*) are frequently associated with these coral gardens [11,54,66,99,100,101]. Bamboo coral seems to play a role in habitat formation, increasing the three-dimensional habitat complexity on flat bathyal bottoms with its candelabrum-like shape. As a passive feeder, its occurrence is often associated with plankton-rich currents which, in turn, favours a high density of prey, such as pandalid shrimps and other crustaceans [102,103,104]. These prey animals attract predators of different trophic levels, such as the abovementioned deep-water red shrimps, bony fishes (e.g., *M. merluccius*, *P. blennoides*, *H. dactylopterus, P. bogaraveo, L. boscii*), and sharks, such as the lesser spotted dogfish (*Scyliorhinus canicula*) and the blackmouth catshark (*G. melastomus*), along with cephalopods (e.g., *I. coindetii*, *T. sagittatus*, *T. eblanae*), all of commercial interest [70,98,105,106,107]. In addition to the important implications as a feeding area for bentho-pelagic species, the arborescent complexity of the colonies could further act as shelter and spawning/nursery sites for several species that can grow to greater sizes than in areas where bamboo coral does not occur [98,104,107,108]. The commercial species associated with bamboo coral account for about 5% of all of the income of the professional fisheries in the Mediterranean [34], with increasing landings—especially in Italy and Spain—the main producers in Europe [55,109].

### 3.2. Open Slope, Hard Bottoms

Coral gardens or coral forests between the shelf break and upper slope are mainly made up of antipatharians and alcyonaceans. Among the former, the most widespread species are *Antipathes dichotoma*, *Parantipathes larix*, *Leiopathes glaberrima_,_* and *Antipathella subpinnata*, which form monospecific or multispecific forests [54,55]. Several commercial fish species are often associated with these antipatharians (e.g., [52,108,110]). Alcyonaceans are present on Mediterranean Sea with hard bottoms with several species, covering a wide bathymetric range. The whip-like gorgonian *Viminella flagellum* and the fan-shaped gorgonian *Callogorgia verticillata* are present in the bathyal zone at depths from 100 to 500 m and from 150 to 1000 m in depth, respectively [54,55]. Off the southwestern coasts of Sardinia, several fish species—some of commercial interest—have been observed hiding among the colonies of *L. glaberrima*, while egg capsules of the shark *S. canicula* have been found on the branches of this antipatharian at depths between 186 and 210 m [52,111]. The presence of egg capsules of this shark on *L. glablerrima* colonies had previously been observed on El Idrissi Bank (Alboran Sea) at 647 m and 452 m [112]. Cau et al. [111] suggested that the coral forest from a representative for southwestern Sardinia represent nursery grounds for *S. canicula*.

In the eastern Ionian Sea, the shark *G. melastomus* and the teleost fish *H. dactylopterus* were the most common fish species caught in the area, characterised by the presence of black coral (*L. glaberrima*) and bamboo coral (*Isidella elongata*) [108]. The shark seems to use the its habitat as a feeding area, and the teleost as a refuge area [108]; *H. dactylopterus* was also found together with other commercial fish species, such as the silver scabbardfish (*Lepidopus caudatus*), and the wreckfish (*P. americanus*) in a coral forest dominated by *L. glaberrima* on the Malta Escarpment (310–315 m) [113].

The fishes that coral zooxanthellate reefs produce in tropical waters account for 17% of animal protein consumed [114]. On the open slope of the Mediterranean Sea, there are still hard bottoms characterised by cold-water coral (CWC) communities, including solitary and colonial zooxanthellae cnidarians [94]. Colonial species have a complex branching morphology, and are habitat formers. The main species, known as white corals, are the colonial species *Madrepora oculata* and *Lophelia pertusa* (recently renamed as *Desmophyllum pertusum*), as well as the solitary coral *Desmophyllum dianthus*. These species have a broad frame-building ability, being able to deposit calcium carbonate and build up durable biogenic substrata. CWCs, as passive suspension feeders, depend on the supply of current-transported particulate organic matter and zooplankton for their trophic requirements. They are preferentially distributed on topographic irregularities on the slope, in canyons and on seamounts, where there are strong currents and the sedimentation rate is low [53,91,115,116,117].

CWC habitats are impacted by fishing due to the occurrence of large sizes and high abundances of commercial species [9,10,13,16,18,58,70,118,119] and references therein]. The presence of corals is generally known to the local fishers, who experience gear damage and losses, although they often fish close to these areas with the aim of obtaining a greater catch and larger specimens of valuable commercial species, such as the deep-water red shrimps (*A. antennatus* and *A. foliacea*) and the European hake (*M. merluccius*). In fact, side-scan sonar and underwater video images show the characteristic seabed scars of otter trawls ploughing through the coral banks [12,82]. Longline is also used in these areas of complex bottom topography, and is not accessible to trawling, so as to catch wreckfish, greater forkbeard, blackbelly rosefish, blackspot seabream, and bluntnose sixgill shark [71,78,81].

CWC habitats provide a suitable ground for larval settlement and juvenile growth of benthic species. They are spawning and nursery areas for vagile and swimming fauna, acting as an EFH for several commercial and non-commercial fish and invertebrate species [12,17,57,71,78,79,80,81,83,84,111].

### 3.3. Canyons

The fishing targeting the deep-water shrimps in the northwestern Mediterranean is carried out on both the slope and the walls of the submarine canyons [11,14,67,120,121,122,123,124].

In the western Mediterranean, *C. conger* and *P. blennoides* are the fish species captured with the highest biomass at the head of the Blanes Canyon [124]. The juveniles of some deep-sea shrimps (e.g., *Plesionika heterocarpus, P. edwardsii, P. gigliolii*, and *P. martia*) and fish (such as *P. blennoides*, *Mora moro*, and *Lepidion lepidion*) are found to be distributed in the benthic intermediate nepheloid layers of the Blanes Canyon, which seems to act as a nursery area for these species [125,126]. From the canyons in the eastern part of the Gulf of Lions, blackmouth catshark and European hake are among the most abundant species [15]. Spawning females of angler and European hake have been more commonly observed within the submarine canyons of Petit-Rhône and Grand-Rhône than on the adjacent open slope [15]. *G. melastomus*, *H. dactylopterus*, and *P. blennoides* are the most frequently observed fish species in French Mediterranean submarine canyons characterised by the presence of CWC species [17]. Discarded fishing gear, including entangled nets and lines, has been observed in the canyons of the western Mediterranean where CWC species thrive [17,127,128,129].

The shark *G. melastomus* and the teleost fish *H. dactylopterus* and *P. blennoides* are the most frequently captured species in the Quirra Canyon (Sardinian waters), where valuable species—such as the European hake and the deep-water red shrimps—are also collected [130].

Eastward, in the Bari Canyon where CWC species thrive, the blackmouth catshark and the teleost fish European conger, blackbelly rosefish, European hake, blackspot seabream, and greater forkbeard have been found to be more abundant in the canyon than in the adjacent area [71,81]. In particular, greater forkbeard showed significantly greater abundance and biomass in the canyon than outside. Blackspot seabream are exclusively caught inside the canyon. A greater number of both smaller and larger individuals of European conger and greater forkbeard are found in the Bari Canyon than on the open slope. Mature females and males are mostly observed in the canyon in all of the most abundant species, indicating the role of the Bari Canyon as a refuge area and an EFH for fish species exploited in the neighbouring fishing grounds [71,83]. Longline remains have been observed in this canyon, which has a complex bottom topography and is not accessible to trawling [97].

Indeed, canyons seem to benefit and support fisheries [14,71,83,131,132], providing spawning and nursery sites as well as refuges for several commercial species [15,17,69,79,80,84,97,122,126,133].

### 3.4. Seamounts

Mediterranean seamounts also host habitat-former species, such as the deep-water glass sponges, sea fans and sea pens, antipatharians—which form large forests up to epibathyal depths—and cold-water corals, such as *Dendrophyllia cornigera* or the white corals *M. oculata* and *D. pertusum*, which thrive at bathyal depths (e.g., [94,134,135]). Due to the topographic and hydrographic conditions allowing the presence of habitat-former species, the seamounts act as biodiversity hotspots, and attract bentho-pelagic fish and migratory species, such as tuna, swordfish, sharks, and cetaceans, as well as several demersal fishery resources [68,76].

The Ulisse Seamount (Ligurian Sea) is a fishing ground for semi-professional and recreational fishermen targeting *P. bogaraveo*, *M. merluccius*, *P. americanus*, pink spiny lobster (*Palinurus mauritanicus*), swordfish (*Xiphias gladius*), and red seabream (*Pagellus acarne*) [136]. In the 1970s, according to the number of hooks employed on the fishing line, the catches were up to several hundreds of kilos, represented by wreckfish, blackspot seabream, bluntnose sixgill sharks, and European conger. A total of 120 wreckfish were caught between 1972 and 1975, before their complete disappearance from the catch data [20].

Accidental by-catch, mainly represented by the large arborescent primnoid anthozoan *Callogorgia verticillata*, provided the first evidence of the existence of coral forests on the summit of the Ulisse Seamount [20].

*A. foliacea* and *A. antennatus* have been reported for other Mediterranean seamounts and banks [68], including the Baronie Seamount located off the northeastern coast of Sardinia [137], representing a site of particular biological and economic interest. Several commercial species—such as the common squid (*Loligo vulgaris*), *P. edwardsii*, *H. dactylopterus*, *A. foliacea*, *A. antennatus*, *G. melastomus*, and *P. blennoides*—have been caught on this seamount [137].

The occurrence of deep-water shrimps (e.g., *A. foliacea*, *A. antennatus*, and *P. martia*), scleractinian corals (e.g., *Caryophyllia calveri*, *Desmophyllum dianthus*), and high densities of other invertebrates was reported in [138] from the Eratosthenes Seamount in the Levantine Basin. In this basin, the presence of sharks (e.g., *G. melastomus* and the spiny dogfish, *Squalus acanthias*), the greater forkbeard, and commercially important deep-sea shrimps (e.g., *P. martia*, *A. foliacea*, and *A. antennatus*) has also been reported for the Turgut Reis Bank [139].

The occurrence of several shark species (e.g., *Prionace glauca*, *H. griseus*, *Cetorhinus maximus*, *Carcharodon carcharias*, *Isurus oxyrhincus*, *Carcharhinus brevipinna*, *Lamna nasus*, *Odontaspis ferox*, and *Sphyrna lewini*) has been detected in the sea area close to the Alcione and Casoni seamounts in the South Tyrrhenian Sea [140].

In the Seco de los Olivos Seamount (western Mediterranean), several commercial species are caught with different fishing techniques, impacting benthic habitats and species [141]: the blue whiting, blackbelly rosefish, silvery pout (*Gadiculus argenteus*), and European hake with otter trawl; *Pagellus* spp., blue whiting, red scorpionfish (*Scorpaena* spp.), and mullets (*Mullus* spp.) using set gillnet; soldier shrimp (*Plesionika* spp.) with traps; and blackspot seabream using bottom longline. In addition, recreational fishing is also carried out on this seamount—mostly on the steeper slopes of the surrounding ridges, targeting the grey grouper (*Epinephelus caninus*) [141].

## 4. Results

### 4.1. Data from FAO Official Statistics

**Figure 3:**
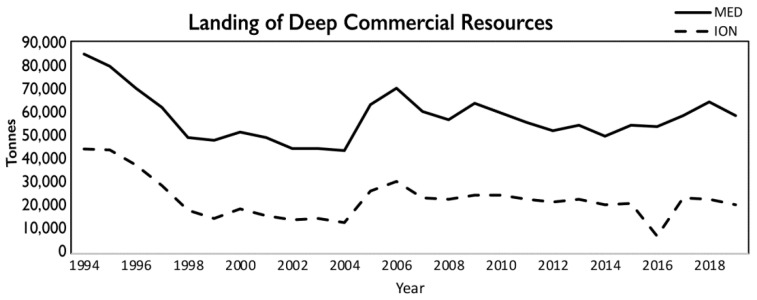
Landings (in tonnes) of deep-sea commercial species in the Mediterranean Sea (black line) and the Ionian Sea (dashed line) in the period 1994–2019, based on official FAO statistics.

During the period 1994–2019, the bulk of the deep-water catches in the Mediterranean Sea was due to the species *M. merluccius*, *P. longirostris*, *A. foliacea*, *A. antennatus*, *N. norvegicus*, *Lophius* spp., *G. melastomus*, *C. conger*, *H. dactylopterus*, *P. bogaraveo*, *Phycis blennoides*, and *P. americanus*. The average total landing for the whole basin was 57,774 (±1857) tonnes per year (7% on average of the Mediterranean total landing; 788,000 tonnes). In the Ionian Sea (FAO subdivision 37.2.2), the average total landing of deep-water species was equal to 23,153 (±1859) tonnes (40% on average of the total landing of Mediterranean deep-water species) (Table 1), thus being the most important area for the exploitation of deep-water resources (Figure 1). A marked fluctuation in landings of the deep-water resources over time has been detected both in the whole Mediterranean and in the Ionian Sea, without any significant trends (Figure 3).

**Figure 4:**
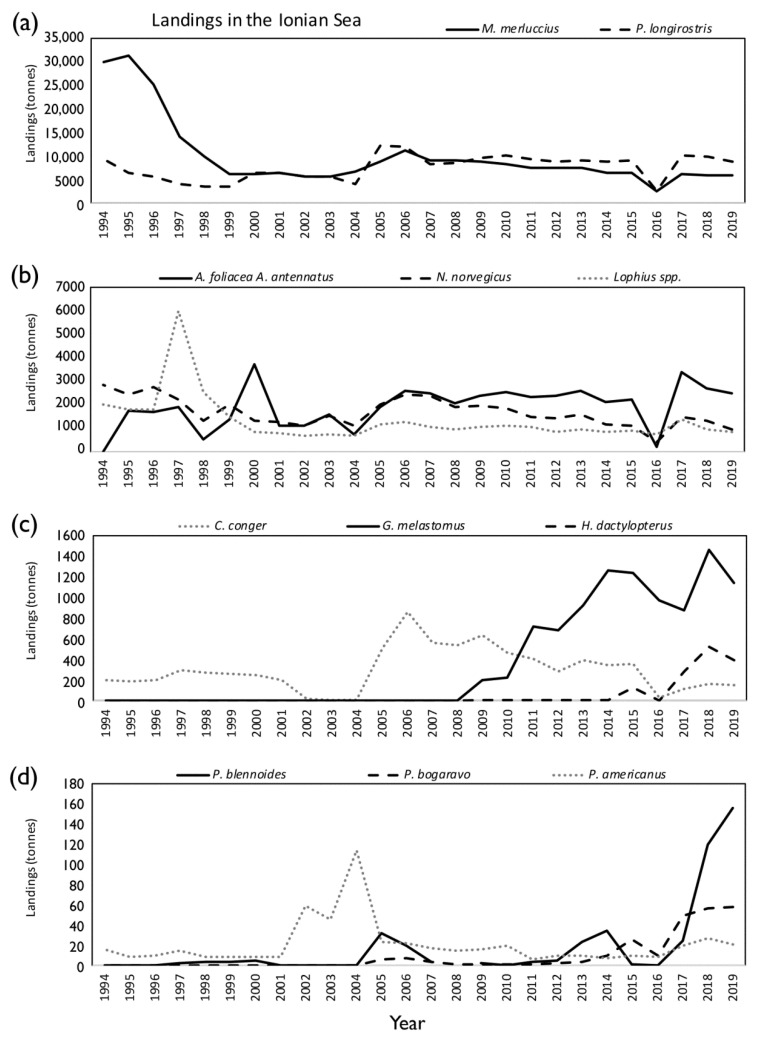
Landings (in tonnes) by deep-sea commercial species—(**a**) *M. merluccius* and *P. longirostris*; (**b**) *A. foliacea*, *A. antennatus*, *N. norvegicus*, and *Lophius* spp.; (**c**) *C. conger*, *G. melastomus*, and *H. dactylopterus*; (**d**) *P. bogaraveo*, *P. blennoides*, and *P. americanus*—caught in the Ionian Sea (FAO subdivisions 37.2.2) during the period 1994–2019.

The main harvested species in the Ionian Sea are the European hake (9865 ± 1445 tonnes and 43% on average), the deep-water rose shrimp (7597 ± 521 tonnes, 33%), the deep-water red shrimps (*A. foliacea* and *A. antennatus*, 1921 ± 177 tonnes, 8%), the Norway lobster (1673 ± 117 tonnes, 7%), and anglers (*Lophius* spp., 1330 ± 207 tonnes, 6%). For *M. merluccius*, *N. norvegicus*, and *Lophius* spp. significant negative temporal trends have been detected (*p* < 0.001, *p* < 0.01, and *p* < 0.05, respectively). In contrast, the landings of the shrimp *P. longirostris* and deep-water red shrimps showed significant positive trends (*p* < 0.05) (Table 1, Figure 4a,b). For the European conger, a clear landing decrease was shown from 2006 (991 tonnes) to 2019 (172 tonnes) (Figure 4c). The exploitation of other deep-water commercial species has increased in this period, as is the case of the blackmouth catshark, with an increase in the landings from a minimum of 193 tonnes in 2009 to a maximum of 1465 tonnes in 2018 (*p* < 0.001). Similarly, *H. dactylopterus* showed a landing increase in the last five years of the time series (*ρ* = 0.584; *p* < 0.01), as did *P. bogaraveo* (*ρ* = 0.681; *p* < 0.001) and *P. blennoides* (*ρ* = 0.542; *p* < 0.01) (Table 1, Figure 4c,d).

**Figure 5:**
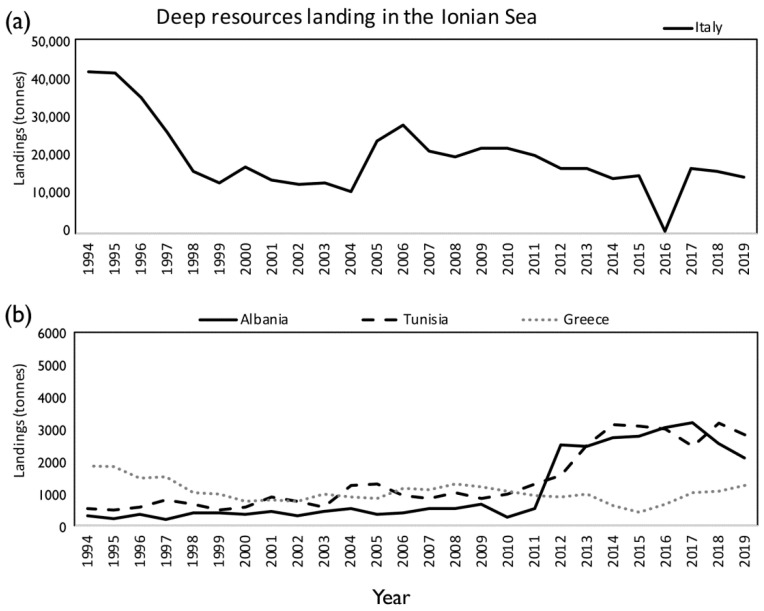
Landings by country ((**a**) Italy; (**b**) Albania, Tunisia and Greece) in the Ionian Sea (FAO subdivisions 37.2.2).

In the Ionian Sea, the Italian fleet shows the highest exploitation of the deep-water resources, with an average landing value of 19,504 tonnes per year in the period 1994–2019, equal to an average of 84% of the total landing from this basin, followed by Tunisia (1418 tonnes; 6%), Albania (1109 tonnes; 5%), and Greece (1062 tonnes; 5%). Marked fluctuations have been observed in the Italian annual landing, with a non-significant decrease, while significant increases have been observed in landings in Tunisia and Albania (*p* < 0.001). A stable trend of landing was shown for Greece (Table 2, Figure 5).

### 4.2. Data from MEDITS Trawl Surveys (NorthWestern Ionian Sea, GSA 19)

**Figure 6:**
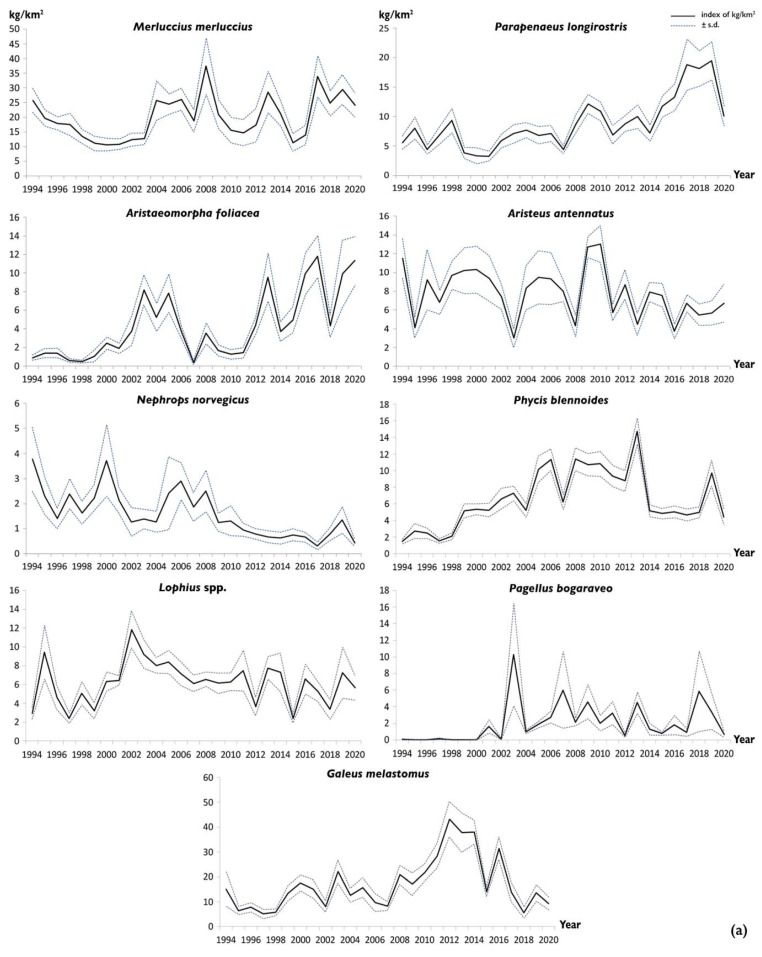

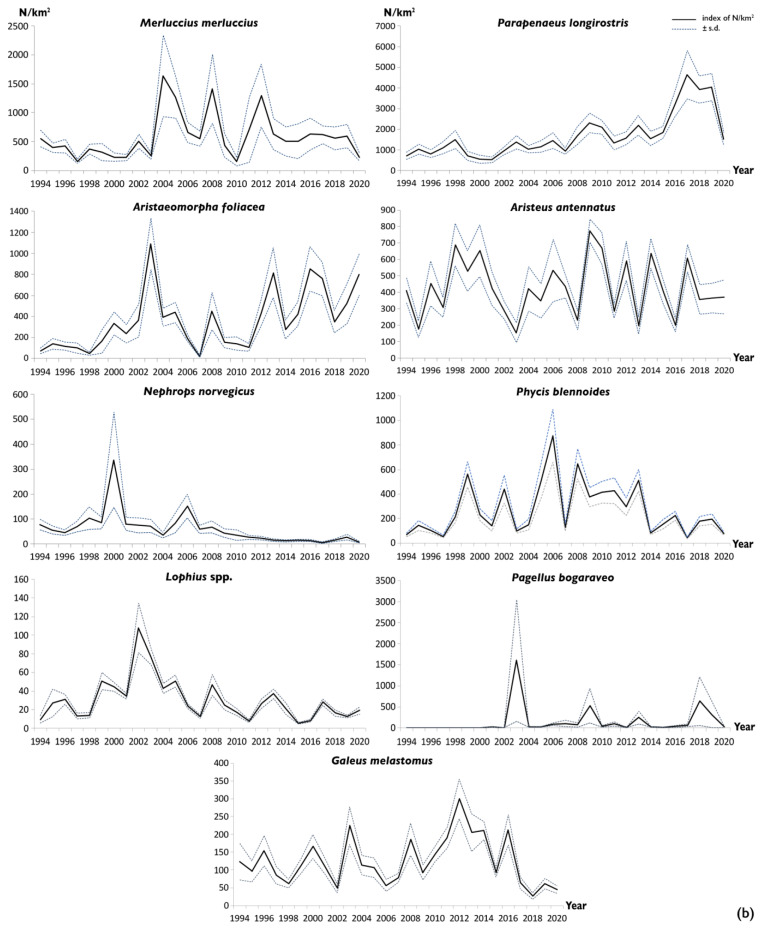
(**a**) Time series of biomass (kg/km^2^) index by species sampled during experimental trawl surveys carried out in the northwestern Ionian Sea from 1994 to 2020. (**b**) Time series of abundance (N/km^2^) index by species sampled during experimental trawl surveys carried out in the nortwestern Ionian Sea from 1994 to 2020.

The European hake and the deep-water rose shrimp are the most abundant species in weight and number, respectively. Highly significant increases in abundance in weight and number over time were detected for the deep-water rose shrimp (*P. longirostris*) (*p* < 0.001, *ρ* = 0.737 and 0.804, respectively), the giant red shrimp (*A. foliacea*) (*p* < 0.001, *ρ* = 0.692 and 0.599, respectively), and the blackspot seabream (*P. bogaraveo*) (*p* < 0.01, *ρ* = 0.538 and *p* < 0.001, 0.634, respectively). A highly significant negative trend for abundance in both weight and number was detected for *N. norvegicus* (*p* < 0.001, *ρ* = −0.766 and −0.785 respectively). A biomass decrease was only shown for *A. antennatus* (*p* < 0.05, *ρ* = −0.391). Fluctuating abundances with no significant trends were observed for the European hake, greater forkbeard, anglers, and blackmouth catshark over the study period (Table 3, Figure 6a,b).

A significant positive relationship was shown between the abundance and SBT in *A. foliacea* (*p* < 0.01, *ρ* = 0.580) and *P. longirostris* (*p* < 0.05, *ρ* = 0.464), while there was a significant negative relationship for *N. norvegicus* (*p* < 0.01, *ρ* = −0.642).

Furthermore, a significant increase in abundance in weight (kg/km^2^) in relation to the decreasing FE was observed for *A. foliacea* and *P. longirostris* (*p* < 0.001, *ρ* = −0.736 and −0.746 respectively), as well as for *P. bogaraveo* (*p* < 0.005, *ρ* = −0.520).

The estimated regression coefficients β_0_ and β_1_ for each model, applied to the log-transformed abundances of *A. foliacea* and *P. longirostris*, are reported in Table 4.

Results highlight the significant effects of both covariates on the abundances of the two species. In particular, for both species, increased abundances significantly depend on a decrease in FE, while significant positive effects are estimated for abundances in relation to the increase in SBT.

**Figure 7:**
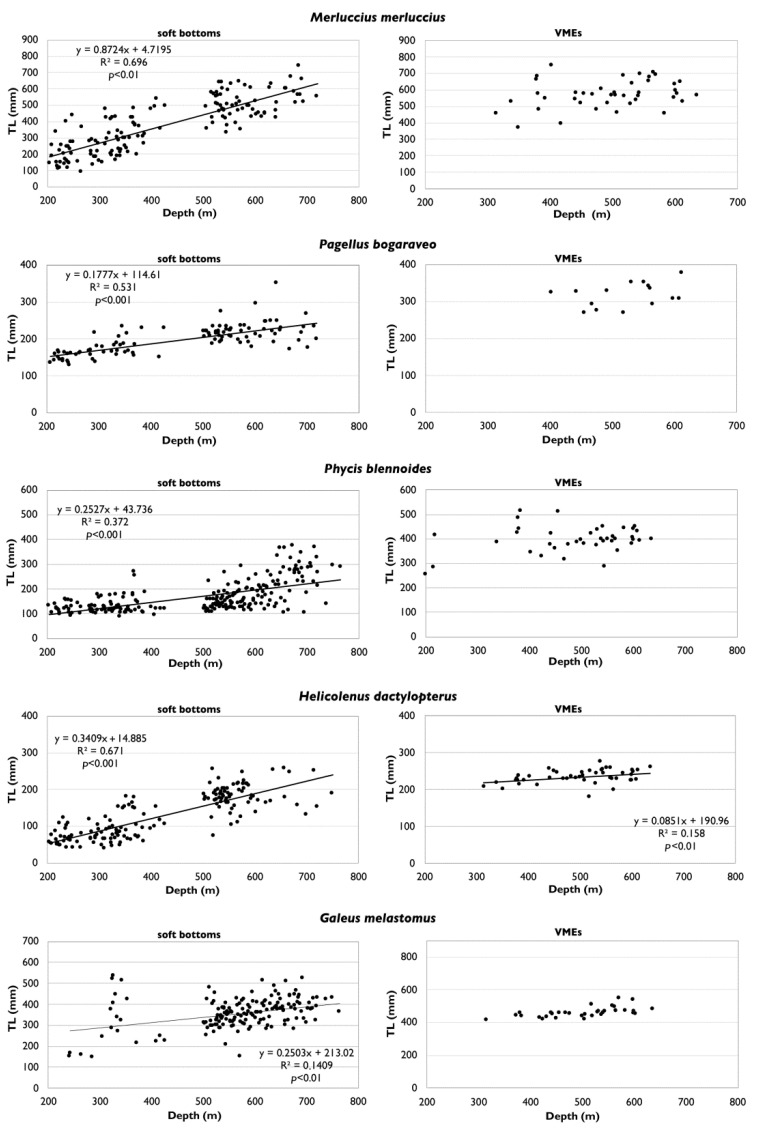
Relationships of total length (TL) with depth of deep-sea species collected on soft bottoms (**left**) and in vulnerable marine ecosystems (VMEs) (**right**) of the central Mediterranean.

**Figure 8:**
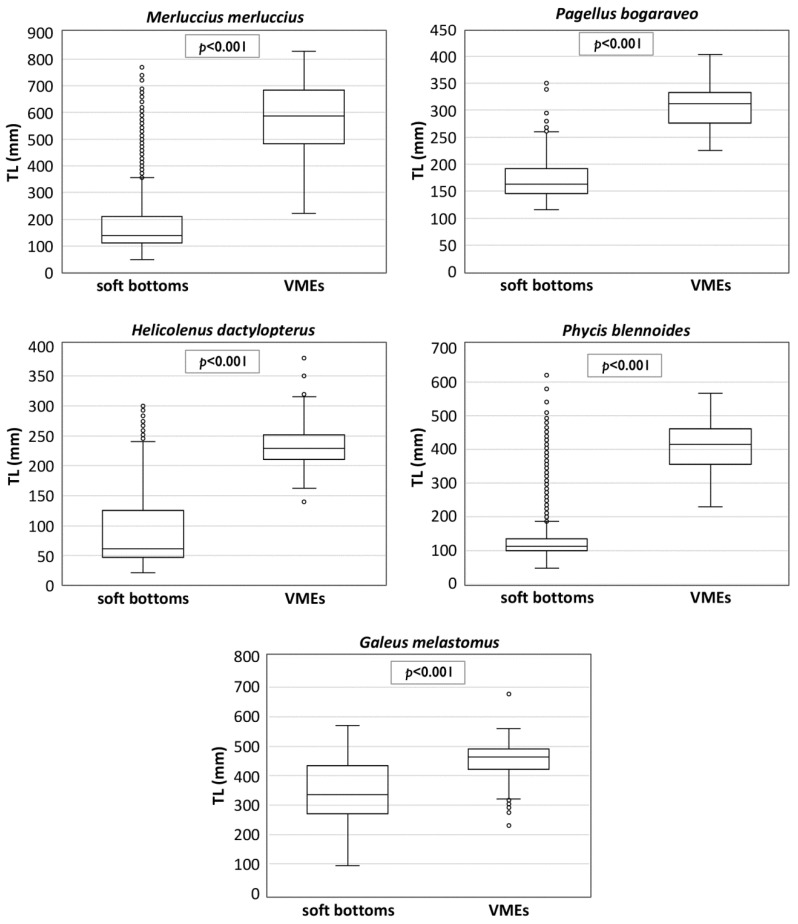
Boxplots of the total length (TL) by species collected on soft bottoms and in vulnerable marine ecosystems (VMEs) of the central Mediterranean.

Regardless of the type of tool used and the type of habitat investigated, the relationships between the sizes and depths show the occurrence of the largest individuals at the greatest depths, with these results being highly significant for all species on soft bottoms, and only for *H. dactylopterus* in VMEs (Figure 7). The sizes were greater in VMEs than on soft bottoms, but this cannot be properly compared due to the different sampling methods and tools (Figure 8).

**Figure 9:**
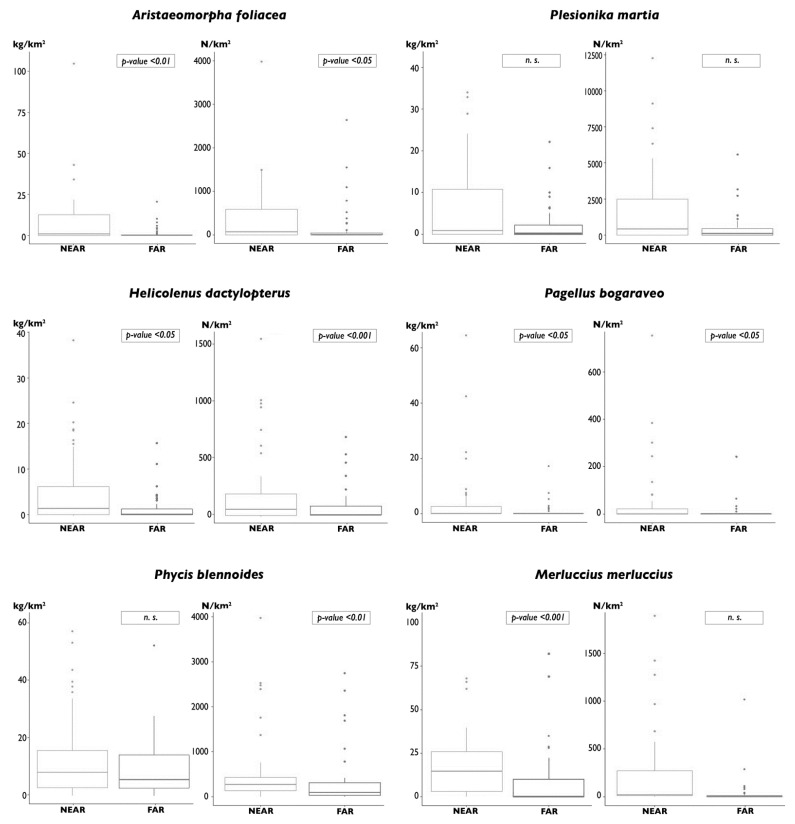
Boxplots of biomass (kg/km^2^) and abundance (N/km^2^) by species collected in two areas of the northwestern Ionian Sea, near and far from the SML fishery restricted area, from 1994 to 2020.

**Figure 10:**
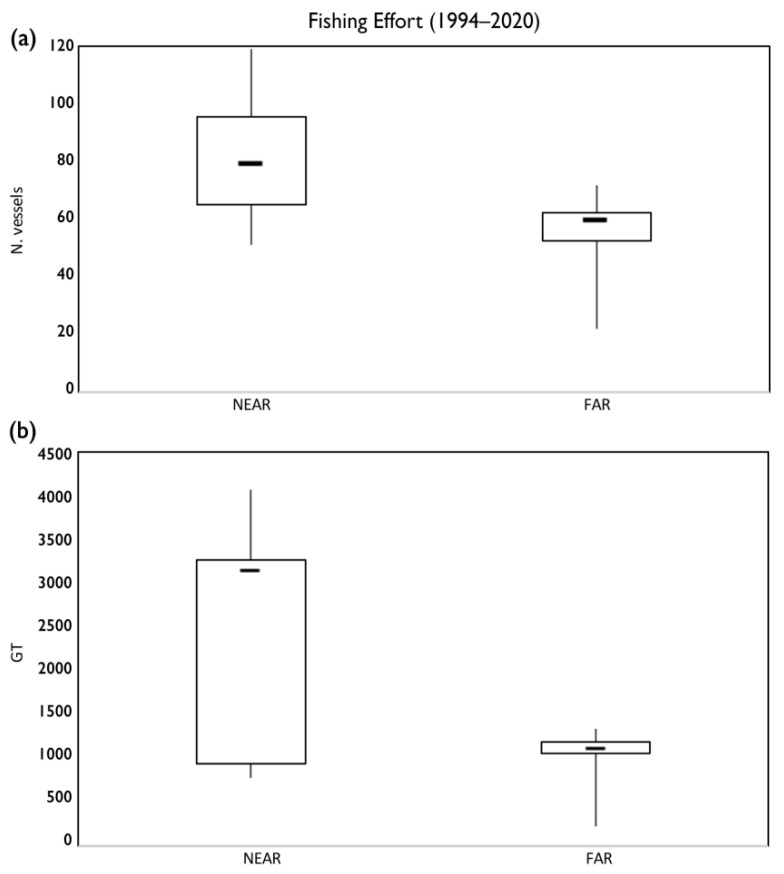
Boxplots of the fishing effort in terms of number (N) (**a**) and gross tonnage (GT) (**b**) of trawl vessels operating near to and far from the SML CWC province during the period 1994–2020.

For all of the deep-sea species considered, the abundances in weight and number for an area near the SML CWC province were greater than those for the area far from this coral province (Figure 9). However, the differences were significant for the abundance in both weight and number of *A. foliacea*, *H. dactylopterus*, and *P. bogaraveo*. The differences between the two areas were significant for the abundance in number of *P. blennoides* and for the abundance in weight of *M. merluccius* (Figure 9). All of these differences are even more significant due to the fact that the fishing effort is significantly greater near the SML CWC province than in the area far from this province in terms of both the number (*p* < 0.001) and the GT of vessels (*p* < 0.05) (Figure 10).

## 5. Discussion

Macrobenthic invertebrates, such as soft and hard corals, contribute to the formation of heterogeneous and complex habitats on slopes, in canyons, and on seamounts, which constitute VMEs. Deep VMEs are hotspots of biodiversity, due to the variety of species they host, and are valuable fishing areas due to the occurrence of large individuals of commercial species and higher catches than in adjacent areas (e.g., [20,55,58,69,70]).

The deep-sea species distributed in the deep-sea habitats and VMEs of the Mediterranean make up 7% of the total quantified landings of approximately 788,000 tonnes [1]. The Ionian Sea is the basin where the largest fraction (about 40%) of the Mediterranean’s deep resources is captured. This basin, in addition to being the deepest in the entire Mediterranean, presents an articulated and complex hydrography and geomorphology of the seabed, with canyons, banks, rocky bottoms, and VMEs where soft and hard coral species thrive (e.g., [81,92,93,94,142,143,144]).

Catches from official FAO statistics show consistency with catches from MEDITS trawl surveys. In both cases, abundance fluctuations were observed for most species. The most abundant deep-sea food resources in the Ionian Sea are the European hake (*M. merluccius*), the deep-water rose shrimp (*P. longirostris*), and the deep-water red shrimps (*A. foliacea* and *A. antennatus*). Other deep-sea food resources are represented by the crustacean *N. norvegicus*, the teleost fish *Lophius* spp., *C. conger*, *H. dactylopterus*, *P. bogaraveo*, *P. blennoides*, and *P. americanus*, and the shark *G. melastomus.*

For some species, significant increases in abundances have been observed, and this may be related to the reduction in fishing effort (e.g., *A. foliacea*, *P. longirostris*, *P. bogaraveo)*, the increase in SBT (e.g., *A. foliacea*, *P. longirostris*), and the presence of refuge areas scarcely accessible to fishing, which constitute VMEs and sites of resource renewal (e.g., *A. foliacea*, *H. dactylopterus*, *P. bogaraveo*, and, to a lesser extent, *P. blennoides* and *M. merluccius*). In particular, adult individuals of *P. bogaraveo* were almost exclusively collected in VMEs between the southwestern Adriatic and northwestern Ionian seas (e.g., [71,78,79,80,145]). The golden shrimp (*P. martia*) was also found to be associated with the presence of corals [145].

Time-series data for the period 1985–2005 reveal a positive relationship of *A. foliacea* and *P. longirostris* abundances with a rise in the water temperature. Inverse relationships of *A. foliacea*, *P. longirostris*, *N. norvegicus*, and *P. blennoides* abundances with FE were also detected [78].

The increase in the abundance of *P. longirostris* observed in the northwestern Ionian Sea has also been reported in all European Mediterranean waters, demonstrating that the abundance of some stock is closely linked to climate change [146]. The increase in temperature observed in recent years may have produced an increase in suprabenthos, e.g., *Lophogaster typicus*, which represents the main prey of the deep-water rose shrimp [146,147]. In addition, as observed in the northern Tyrrhenian, high temperatures and low wind circulation negatively affect the recruitment of *M. merluccius.* Hake juveniles prey upon *P. longirostris* juveniles [148], so the lower predation pressure could have further enhanced the recruitment success of the shrimp [146,149]. The significant increase in abundance detected for *A. foliacea* is consistent with observations in the eastern Ionian Sea and southern Adriatic Sea [150]. However, for both *P. longirostris* and *A. foliacea*, the reduction in FE may have influenced the increase in their abundances.

Regarding *A. antennatus*, in the western Mediterranean it was observed that this deep-water shrimp seems to prefer relatively cold temperatures (13.1–13.2 °C) and relatively salty waters (>38.5) with low currents and moderate variability [123]). However, the occurrence of *A. antennatus* appears to be driven in a nonlinear manner by environmental conditions, including local temperature [123].

Times-series data regarding the abundance of *M. merluccius* in the northwestern Ionian for the period 1985–2005 reveal a positive relationship with the NAO index [78]. Recently, it has been observed that environmental factors can affect the spatiotemporal distribution pattern of the European hake throughout the Mediterranean basin [151]. In particular, high predicted biomass levels were observed especially at 200 m and between 14 and 18 °C, highlighting the preference of the species for colder waters. Moreover, the high biomass of this teleost fish has been correlated with the presence of nursery areas in many Mediterranean areas, some of which have been identified along the northwestern Ionian and in the southwestern Adriatic [152,153,154,155], connected to VMEs [81,83]. In this respect, the effect of the presence of the SML VME has been detected for *M. merluccius* and other deep-water species, in agreement with previous investigations in the central Mediterranean (e.g., [12,71,81,83,85,98]). Indeed, the SML CWC province has an effect on the abundance of deep-water resources, while also contributing to the spillover of individuals of commercial species exploited in the surrounding fishing grounds, subjected to a greater fishing pressure than in other areas of the northwestern Ionian Sea.

The most abundant species captured on the muddy bottoms, both by commercial fishing (FAO data) and during trawl surveys (MEDITS data), are also those most frequently caught in sensitive habitats and VMEs. Most of these species exhibit a bigger–deeper pattern, both on muddy bottoms—where larger individuals are generally caught by trawling—and in sensitive habitats and VMEs, partially protected from fishing due to the roughness of the seabed and the presence of rocks and hard corals. The largest individuals distributed in deep waters and in VMEs represent the adult fraction of the stocks. These are breeder individuals that often concentrate in VMEs, making them EFHs, since these ecosystems act as spawning and nursery areas [12,57,71,81,98], contributing to the renewal of the stocks on fishing grounds [83]. Both the spillover (i.e., active movement of juveniles and adults into fished areas) and larval seeding (i.e., the dispersal of eggs and larvae in fished areas) may contribute to the renewal of stocks in neighbouring fishing grounds. All of the species examined in this study carry out their life cycles on the bathyal bottoms by distributing themselves between muddy bottoms and refuge areas represented by VMEs, but only in these ecosystems is their vulnerability to fishing activities greatly reduced. In fact, models developed using data from trawl surveys carried out on muddy bottoms provide indications of overfishing status for most of the species examined in the present study (e.g., [156]).

Species with a wide bathymetric distribution, such as anglers, have not shown any significant trend over time. Norway lobster, for both types of data, showed a decrease in abundance over time. This may be due to the fact that this species needs soft bottoms to dig burrows in the mud, which are also the most subjected to trawling. Another explanation deals with the negative relationship between Norway lobster biomass and SST, with the lowest indices associated with high temperatures [146]. The decreasing abundance related to higher temperatures is due to the reduction in the organic matter flux resulting from the decreased rainfall and river discharge, which influences benthic feeders and predators such as the Norway lobster [146,147].

Several deep-water species also represent an important feeding resource for several species of odontocetes inhabiting the offshore areas of the northwestern Ionian Sea [157,158,159,160]. Bathyal bentho-pelagic squids are fundamental prey of the Risso’s dolphin (*Grampus griseus*) and the sperm whale (*Physeter macrocephalus*). Myctophids and demersal species, such as *M. merluccius*, are hunted by the striped dolphin (*Stenella coeruleoalba*) [161,162,163,164]. The trophic interactions between cetaceans and deep-water prey contribute to the recycling of energy and matter in the pelagic domain and coastal areas. Odontocetes can increase the CO_2_ absorption capability of phytoplankton, thus playing a critical role in climatic regulation [165]. Thus, the management of deep sensitive habitats and their species contributes to ensuring the stability of several biological components that play a critical role in ecosystem functioning [164].

The increase in landings observed for Tunisia and Albania could be due to the fact that there are still deep areas that are largely unexploited by fishing activity. Moreover, here, the fishing pressure is lower than that along the Italian coasts of the central Mediterranean.

The VMEs are often impacted by commercial fishing (e.g., [17,20,64,68,69,82,98,103,104,106,107,127,129]). An overview of available information on the incidental catch of VME indicator taxa from fishery-dependent and fishery-independent surveys has been recently provided by Chimienti et al. [55]. Bottom trawls represent the most impactful fishing practice to deep soft-bottom VMEs, followed by longlines, gillnets, pots, and traps.

Other human activities—including oil and gas exploration and exploitation, pollution, marine litter, ocean acidification, and climate change—are also harmful to VMEs and their biodiversity on the open slopes, canyons, and seamounts (e.g., [14,64,69,126,166,167,168]). In addition, biodiversity loss affects ecosystem services (ESs) and impairs the ocean’s capacity to provide food, maintain water quality, and recover from disturbance [169,170,171,172]. ESs are the benefits that humans derive, either directly or indirectly, from the functions of ecosystems [173]. The Millennium Ecosystem Assessment (MA) [174] estimates that 60% of global ecosystem services are degraded or are being managed unsustainably. As biodiversity is lost and ecosystems are degraded, the biocapacity of the planet to support living organisms decreases. As biocapacity decreases, there are diminishing resources available to support a growing human population [175]. These considerations also concern the Mediterranean and its VMEs that provide several ESs [54] and, as noted above, many of them are in danger due to anthropogenic impacts.

The habitat structured by macrobenthic invertebrates in VMEs is a supporting ES that provides organisms—including those of commercial species—with suitable physical and chemical features, food and spatial resources, places for courtship, mating, and spawning, breeding sites and nurseries, places to hide from predators, and refuges to escape from adverse environmental conditions (e.g., [38,45,58,72,176,177]). The decline of the habitat can cause negative effects on the species that use it for bioecological processes, affecting community composition and ecosystem functioning [178,179].

A strategy based on ecosystem-based fishery management (EBFM) was adopted by the EU Common Fishery Policy (CFP) for fishery management, with the overall objective of sustaining healthy marine ecosystems and the fisheries that they support [180]. This implies sustainable management not only of the commercial stocks, but also of the whole environmental system that supports their production, including the importance of the economic and social dimensions.

Worldwide, fish consumption has grown enormously since 1961, surpassing even that of several species of the most common terrestrial animals in the human diet. Thinking about the fact that food innovation and sustainability are mainly based on systems where it is produced and systems by which food is collected, there is an urgent need to protect deep-sea habitats and VMEs by promoting the use of sustainable fishing systems.

Many governments have made international commitments to the conservation of marine biodiversity. In particular, since the adoption of the Convention on Biological Diversity (CBD) in 1992, biodiversity considerations in relation to the management of fisheries and aquaculture have been focused on policies and actions for the conservation of threatened species and vulnerable habitats. Within the CBD context, the scientific criteria to identify ecologically and biologically significant marine areas have been established [181], with the aim of defining management measures that ensure the conservation of the biodiversity of these areas. The process in the Mediterranean was started by a regional workshop in 2014 [182], and culminated at the CBD COP 12 with the endorsement of 15 ecologically and biologically significant marine areas, recognising the biological and ecological significance of deep-sea habitats in the Mediterranean [64]. Following specific UNGA resolutions [183,184,185], similar criteria were adopted by the FAO [46] to identify VMEs and to develop international guidelines for the management of deep-sea fisheries in the high seas, in order to ensure the protection of certain groups of species and habitats from significant adverse impacts (SAIs) caused by fisheries.

The Habitats Directive (92/43/EEC) [186] considers the CWC biotope as the habitat type “1170 Reefs”, for which measures should be taken to maintain and restore a conservation status for this type of habitat. As part of the Barcelona Convention, the protocol concerning the specially protected areas and biological diversity adopted in 1995 is a tool for implementing the CBD, since it aims to protect and conserve biodiversity in valuable areas and species in the Mediterranean Sea. More recently, the Marine Strategy Framework Directive [187] aims to ensure that the collective pressure of human activities on the environment is kept within levels compatible with the achievement of good environmental status. This can also be achieved through the creation of marine protected areas (MPAs) focused on achieving a balance between sustainable fisheries and other human activities and habitat conservation. Although several conservation initiatives have been developed with the aim of protecting threatened hotspots of marine biodiversity, the marine protected areas (MPAs) that have been designated in deep waters are very limited [64,188,189].

During the last few decades, the Mediterranean deep-sea habitats have been protected through the institution of fishery restricted areas (FRAs), established by the General Fishery Commission for the Mediterranean and Black Sea (GFCM) with the aim of protecting VMEs and/or essential fish habitats (EFHs). To date, 10 FRAs have been established by the GFCM, including 1 large deep-water FRA below 1000 m. Trawling is forbidden in areas deeper than 1000 m in depth throughout the Mediterranean Sea. However, this conservation measure is not enough, considering that most of the coral habitats known so far are present within this depth limit. For this reason, the limitation of trawling up to 800 m depth would be more effective for the conservation of deep-sea coral habitats [54,64]. The FRAs aim to protect EFHs and/or sensitive habitats of high ecological value, such as VMEs, from any SAIs of fishing activities [46]. In particular, only two of the existing FRAs in the Mediterranean Sea have been created to target the conservation of a CWC habitat—namely, the *Lophelia* Reef off Santa Maria di Leuca (Italy, Ionian Sea) [82] and, more recently, the Bari Canyon in the southern Adriatic Sea (recommendation GFCM/44/2021/3) [133]. Three FRAs in the Strait of Sicily (northeast and northwest Malta) and one in the Gulf of Lion include some CWC sites, but they have been created to manage fishing stocks; thus, trawling is present there, albeit somewhat regulated. The Jabuka/Pomo Pit FRA aims to protect EFHs and an unquantified sea pen field [190], while the Eratosthenes Seamount FRA targets the protection of peculiar geological formations (with only a few specimens of solitary scleractinians recorded) [138], and the Nile Delta FRA is characterised by the presence of chemosynthetic fauna [54]. Trawling and dredging are forbidden in the FRAs for the conservation of VMEs, while they are regulated in those for the management of EFHs. Bottom longlining can be allowed—often in a buffer zone and under authorisation—depending on the regulations of the single FRA, while artisanal fishing practices are usually not performed in offshore areas, such as in the existing FRAs. These FRAs are currently isolated, while a desirable network of FRAs is a long way from being created. This network should be established in the pathway of the Mediterranean water mass circulation in order to connect the different FRAs all over the basin by means of larval dispersal [53,83,129].

As part of goal 14 (life below water) of the 2030 Sustainable Development Agenda [191], all of the targets regard VMEs, and specifically targets 14.2 (protect and restore ecosystems), 14.3 (reduce ocean acidification), and 14.4 (sustainable fishing). More recently, the EU Biodiversity Strategy for 2030 [192] reports that biodiversity is also seen as essential for safeguarding EU and global food security; its loss threatens our food systems, putting our food security and nutrition at risk.

## 6. Conclusions and Recommendations

In the EU, at least 30% of the land surface and 30% of the sea should be protected, and for areas with high value or potential for biodiversity and, therefore, greater vulnerability, more stringent protection is needed.

Regarding the deep-sea sensitive habitats and VMEs, the implementation of a network of protected or fishing-restricted areas represents a fundamental measure to guarantee the proper conservation of the sites known in the Mediterranean Sea. A network of protected areas (mostly MPAs and FRAs) would satisfy both the conservation of vulnerable habitats and the management of fishery resources according to the EBFM [63]. In this respect, after the past and recent establishment of the FRAs in the SML CWC province and Bari Canyon, respectively, appropriate spatial measures aimed at preserving the ecological function of *I. elongata facies* identified in the southern Adriatic should be adopted [98]. The Bari Canyon, *I. elongata facies*, and SML CWC province are in the path of the flow of the dense-water masses that pour from the southern Adriatic into the northern Ionian [53,81,92,94,97,129,193]. This stream of water masses connects the deep-sea coral communities distributed along the Apulian margin, which represent a network of refuge/renewal areas of fishery resources [81] that needs coherent conservation measures and management strategies according to the EBFM [53,54,55].

In the southern Adriatic, the activity of trawler fleets is more concentrated on the continental shelf and on the upper part of the slope, with target species including the European hake, red mullet, spottail mantis shrimp, deep-water rose shrimp, and Norway lobster [194]. This explains the persistence of *I. elongata* in the southern Adriatic [98], and would suggest that the protection measure to be adopted would be accepted by the fishermen. If not accepting an area closure measure, other management approaches—such as encounter protocols with associate thresholds—can be developed and implemented according to the FAO’s International Guidelines for the Management of Deep-Sea Fisheries [46,195], in order to ensure the protection of *I. elongata* from SAIs. In addition, the use of onboard observers and the correct adoption of digital logbooks could be applicable to trawl fishing vessels, as well as to most of the deep-sea benthic longlining vessels, which must be equipped with vessel monitoring by satellite systems (VMSs) and/or automated identification systems (AISs). The management and control of the many small artisanal fishing boats, which use gillnets and trammel nets in shallower waters, could be achieved through the designation of landing points, obligations of notice of arrival in port, and control of landings [54].

Through blue growth (BG), the European Union seeks to meet human needs—such as food and energy—in a sustainable way, by creating new jobs and new sources of growth while safeguarding biodiversity and protecting the marine environment, thus preserving the services that healthy and resilient marine and coastal ecosystems provide [196]. In this respect, marine spatial planning [187] consists of a public process of analysing and allocating the spatial and temporal distribution of human activities in marine areas to achieve ecological, economic, and social objectives, reducing conflicts and creating synergies between different activities, while protecting the environment by assigning protected areas, calculating impacts on ecosystems, and identifying opportunities for multiple uses of space. The use of an ecosystem for economic returns and social benefits must be done in a way that minimises negative impacts. If an ecosystem is degraded, the ESs that are derived from it will also be modified, including those concerning the availability and safety of food.

The good governance of an ecosystem requires, first of all, knowledge and monitoring of its condition. The central Mediterranean is an area with a complex hydrology and geomorphology, rich in biodiversity and biological resources, requiring greater knowledge and continuous monitoring of sensitive habitats and VMEs, as well as their associated biotic components, which can represent food resources [64,76]. Danovaro et al. [197] used expert elicitation (1155 deep-sea scientists consulted and 112 respondents) to indicate a wide consensus that monitoring should prioritise large organisms (that is, macro- and megafauna) living in deep waters and in benthic habitats, whereas monitoring of ecosystem functioning should focus on trophic structure and biomass production. They suggested that deep-sea conservation efforts should focus primarily on VMEs and habitat-forming species.

This is of particular importance in relation to the growth of human populations and the expansion of activities regarding the deep-sea environment, and for which the involvement of stakeholders with an interest in the deep-sea will be necessary, together with mechanisms that promote wide participation at the national and international levels, and that ensure conservation and long-term effective ecosystem-based fishery management measures.

## Figures and Tables

**Table 1:** Total landing (in tonnes and %), with mean and standard error (SE), in the Mediterranean (MED) and Ionian Sea (ION), and by species, in the period 1994–2019. *ρ* = Spearman’s rank correlation coefficient; *p* = *p*-value; n.s.: non-significant values.

	Landing Tonnes			
	Mean ± SE	%	Spearman’s *ρ*	*p*
MED	57,774 ± 1857	-	−0.059	n.s
ION	23,153 ± 1754	40%	−0.210	n.s
*M. merluccius*	9865 ± 1445	43%	−0.637	<0.001
*P. longirostris*	7597 ± 521	33%	0.426	<0.05
*A. foliacea* and *A. antennatus*	1921 ± 177	8%	0.446	<0.05
*N. norvegicus*	1673 ± 117	7%	−0.588	<0.01
*Lophius* spp.	1330 ± 207	6%	−0.462	<0.05
*C. conger*	296 ± 41	1%	0.079	n.s
*G. melastomus*	373 ± 101	2%	0.853	<0.001
*H. dactylopterus*	52 ± 26	>0.2%	0.584	<0.01
*P. blennoides*	17 ± 7	>0.2%	0.542	<0.01
*P. bogaraveo*	9 ± 3	>0.2%	0.681	<0.001
*P. americanus*	20 ± 4	>0.2%	−0.060	n.s

**Table 2:** Total landing (in tonnes and %), with mean and standard error (SE), by Ionian countries in the period 1994–2019. *ρ* = Spearman’s rank correlation coefficient; *p* = *p*-value; n.s.: non-significant *p*-values.

	Landing (Tonnes)			
Country	Mean ± SE	%	Spearman *ρ*	*p*
Italy	19,504 ± 1792	84%	−0.353	n.s.
Greece	1062 ± 67	5%	−0.332	n.s.
Malta	48 ± 7	<0.2%	0.760	<0.001
Tunisia	1418 ± 192	6%	0.902	<0.001
Albania	1109 ± 215	5%	0.856	<0.001

**Table 3:** Mean values of biomass (kg/km^2^) and density (N/km^2^) indices with standard deviation (s.d.), computed by species on the 1994–2020 time series of experimental trawl surveys carried out in the northwestern Ionian Sea. *ρ* = Spearman’s rank correlation coefficient; *p* = *p*-value; n.s.: non-significant *p*-value.

		kg/km^2^			N/km^2^	
	Mean ± s.d.	Spearman *ρ*	*p*	Mean ± s.d.	Spearman *ρ*	*p*
*Merluccius merluccius*	19.98 ± 7.40	0.338	n.s.	585 ± 385	0.336	n.s.
*Parapenaeus longirostris*	8.87 ± 4.50	0.737	<0.001	1697 ± 1086	0.804	<0.001
*Aristaeomorpha foliacea*	4.34 ± 3.63	0.692	<0.001	362 ± 284	0.599	<0.001
*Aristeus antennatus*	7.77 ± 2.67	−0.391	<0.05	427 ± 173	−0.034	n.s.
*Nephrops norvegicus*	1.59 ± 0.90	−0.766	<0.001	61 ± 65	−0.785	<0.001
*Phycis blennoides*	6.59 ± 3.47	0.313	n.s.	272 ± 211	0.052	n.s.
*Lophius* spp.	7.77 ± 2.67	0.023	n.s.	427 ± 173	−0.326	n.s.
*Pagellus bogaraveo*	2.06 ± 2.44	0.538	<0.01	145 ± 333	0.634	<0.001
*Galeus melastomus*	16.96 ± 10.54	0.344	n.s.	125 ± 67	−0.085	n.s.

**Table 4:** Effect of fishing effort and temperature covariates on *P. longirostris* and *A. foliacea* abundance indices in biomass (kg/km^2^) and density (N/km^2^). Estimate = Estimated coefficient; SE = Standard Error; *t* = *t*-value; *p* = *p*-value.

		Effect	Estimate	SE	*t*	*p*
***P. longirostris***	Biomass (kg/km^2^)	Intercept	4.33	0.42	10.29	<0.001
		Fishing effort	−0.01	0.01	−5.30	<0.001
		Intercept	−3.36	2.16	−1.56	0.136
		Temperature	0.36	0.14	2.55	0.02
	Density (N/km^2^)	Intercept	9.9	0.52	19.10	<0.001
		Fishing effort	−0.01	0.01	−4.97	<0.001
		Intercept	0.97	2.57	0.38	0.712
		Temperature	0.42	0.17	2.49	0.022
***A. foliacea***	Biomass (kg/km^2^)	Intercept	5.04	0.72	6.97	<0.001
		Fishing effort	−0.02	0.01	−5.14	<0.001
		Intercept	−9.48	3.39	2.80	0.011
		Temperature	0.72	0.23	3.21	0.005
	Density (N/km^2^)	Intercept	8.39	0.81	10.32	<0.001
		Fishing effort	−0.01	0.003	−3.18	0.005
		Intercept	−1.61	3.38	−0.48	0.639
		Temperature	0.5	0.22	2.21	0.04

## Data Availability

The data used in this study were collected under the Data Collection Framework (DCF), supported by the Italian Ministry of Agriculture, Food and Forestry Policy (MiPAAF) and by the European Commission (EU Regulations 1004/2017). Data provided are owned by the Italian Ministry of Agriculture, Food and Forestry Policy (MiPAAF).
